# Resignation of Dr. Joseph Head

**Published:** 1893-03

**Authors:** 


### RESIGNATION OF DR. JOSEPH HEAD.

We regret to be obliged to announce that Dr. Head has, by press of work in other directions, felt compelled to tender his resignation as assistant editor of this journal. We part with him with great regret, and trust that his present separation from editorial work may be only temporary.

Dr. George W. Warren, of Philadelphia, author of a "Compend of Dental Pathology and Dental Medicine," and chief of clinics of the Pennsylvania College of Dental Surgery, was elected to fill his place. We have every confidence that Dr. Warren will ably second the efforts of the editor-in-chief to increase the value of the Journal, by extending its work and adding interest to its pages.

